# Serum Lactate Dehydrogenase Level as a Prognostic Factor for COVID-19: A Retrospective Study Based on a Large Sample Size

**DOI:** 10.3389/fmed.2021.671667

**Published:** 2022-01-04

**Authors:** Yihui Huang, Liang Guo, Jiwei Chen, Meng Wu, Chao Zhang, Zeming Liu, Jinpeng Li, Kun Li, Zhongwei Xiong, Qian Wu, Zhengwei Li, Kuan Luo, Weiwei Yuan, Xiaohui Wu

**Affiliations:** ^1^Department of Plastic Surgery, Zhongnan Hospital of Wuhan University, Wuhan, China; ^2^Department of Thyroid and Breast Surgery, Zhongnan Hospital of Wuhan University, Wuhan, China; ^3^Department of Ultrasound, Zhongnan Hospital of Wuhan University, Wuhan, China; ^4^Department of Cardiovascular Surgery, Tongji Medical College, Union Hospital, Huazhong University of Science and Technology, Wuhan, China; ^5^Department of Plastic Surgery, Tongji Hospital, Tongji Medical College, Huazhong University of Science and Technology, Wuhan, China; ^6^Department of Hepatobiliary and Pancreatic Surgery, Zhongnan Hospital of Wuhan University, Wuhan, China; ^7^Department of Neurosurgery, Zhongnan Hospital of Wuhan University, Wuhan, China; ^8^Department of Neurosurgery, Wuhan Puren Hospital, Wuhan, China; ^9^Department of Plastic and Cosmetic Surgery, Shenzhen People's Hospital (The Second Clinical Medical College, Jinan University, The First Affiliated Hospital, Southern University of Science and Technology), Shenzhen, China

**Keywords:** COVID-19, lactate dehydrogenase (LDH), SARS-CoV-2, prognostic factor, Leishenshan Hospital

## Abstract

**Background:** In this study, we investigated the relationship between serum lactate dehydrogenase (LDH) level and disease progression and prognosis of patients with COVID-19.

**Methods:** We retrospectively reviewed the information of 1,751 patients with COVID-19 from Leishenshan Hospital in Wuhan, China. Univariate and multivariate Cox regression analyses as well as Logistics regression analyses, and Kaplan-Meier curves were used to determine the association between LDH levels and the prognosis of COVID-19 patients.

**Results:** LDH was an independent risk factor for in-hospital death no matter it was taken as classified variable and continuous variable (all *P* = 0.001) but not for severe or critical illness status. The Kaplan-Meier curves for LDH level showed that an elevated level of LDH was associated with in-hospital death.

**Conclusions:** In patients with COVID-19, the increased LDH level is associated with a higher risk of negative clinical prognosis and higher mortality. This will provide a reference for clinicians and researchers to understand, diagnose, and treat patients with COVID-19. Further prospective studies with larger sample sizes are needed to verify these findings.

## Introduction

The world is currently experiencing a major coronavirus disease 2019 (COVID-19) pandemic (1–3). Although COVID-19 can cause severe illness, the case fatality rate is relatively low (4). As of 22 April 2020, more than 2,500,000 cases were reported worldwide, with more than 170,000 deaths.

Leshenshan Hospital is hosted by Zhongnan Hospital and is a temporary, specialized 1,600-bed hospital designated for the treatment of patients with COVID-19. From February 8, 2020 to April 15, 2020, 1,880 patients with confirmed COVID-19 were admitted. Lactate dehydrogenase (LDH) is one of the enzymes of the glycolytic pathway that catalyzes the conversion of pyruvate to lactate with concurrent conversion of reduced nicotinamide adenine dinucleotide (NADH) to nicotinamide adenine dinucleotide (NAD) (5). Elevated LDH levels have been shown to be associated with more severe disease and increased mortality in multiple diseases (5–7).

Clinicians and researchers have been making efforts to understand and cure COVID-19; however, knowledge of its pathogenesis is limited (8–10). In our study, we investigated the effect of serum LDH levels on the disease progression and prognosis of patients with COVID-19. There is usually a normal range for the measurement of LDH in clinical application. The study group assignment in this study was generated based on the normal range of LDH level but we also took LDH as a continuous variable when conducting analyses so that we can intuitionally detect the relation between LDH level and the prognosis of COVID-19 patients.

## Materials and Methods

### Study Design and Patients

We conducted a retrospective cohort study of 1,880 patients with laboratory-confirmed SARS-CoV-2 infection, who were admitted to Leishenshan Hospital in Wuhan, China, with COVID-19 between February 9 and March 18, 2020. The medical records of these patients were reviewed by two experienced physicians, and detailed information on patient demographics, clinical features, laboratory test results, computed tomography (CT) images, and treatment were extracted. A total of 129 patients who did not have an LDH test or whose LDH test results were missing were excluded, leaving 1,751 patients for the analysis. Of these patients, 1,653 had an LDH level within the normal range (125–343 U/L), 43 had a low LDH (<125 U/L), and 55 had an elevated LDH (>343 U/L). Considering the clinical implications of the LDH results, patients with a normal or decreased LDH level were assigned to one group and compared with the 55 patients with an elevated LDH level. LDH was also taken as a continuous variable when conducting the analyses for the prognosis of COVID-19 patients. All the laboratory findings were baseline data including LDH level.

### Definitions

The primary outcome in this study was the occurrence of death during the period of hospitalization. The illness status was defined according to the seventh edition of the Chinese management guideline for COVID-19 published by the Chinese National Health Commission (11). We acquired records of the illness status on admission and the highest level of illness status of patients during their hospitalization. The latter was also used as an outcome in this study. Mild and common cases were assigned in one group while severe and critical cases were combined into one group when illness status was used as an outcome in analysis. The survival time in this study was defined as the period from the day that patients on admission to the day deaths occurred or follow-up stopped and it was described as “follow-up days.” An axillary temperature over 37.3°C was defined as fever. A semi-quantitative score system based on the results of the CT images was generated to evaluate the pulmonary lesions of COVID-19 patients. Each of the image characteristics including ground-glass opacities, reticulation or cords change, consolidation, and pleural effusions were assigned 1 point. Score 1 was the sum of the points mentioned above. Score 2 was assigned based on the area of the lung lobes involved: no involvement (0 points); <25% involvement (1 point); 26–50% (2 points); 51–75% (3 points); >75% (4 points). The total score was equal to the sum of score 1 and score 2.

### Ethics Approval and Patient Consent

This study obtained the approval of the Research Ethics Commission of the Zhongnan Hospital of Wuhan University (approval number: 2020074). The requirement for informed consent was waived because the study was retrospective.

### Statistical Analysis

All the statistical analyses were performed using SPSS Version 23.0 (IBM Corp, Armonk, NY, USA). Comparisons between the low/normal and elevated LDH level groups for categorical data were performed using the chi-square test or Fisher's exact test if the number of observations was limited. Comparisons of continuous variables were performed using independent group *t*-tests when the data were normally distributed, or Mann-Whitney U test when the data were not normally distributed. Univariate and multivariate Cox regression analyses were conducted for investigating the relation between in-hospital death and LDH level, while Logistics regression analyses were generated for detecting the relation between illness status and LDH level. Factors which were significant associated with primary outcomes in univariate analyses were selected into adjustment when conducting multivariate analyses. For intuitionally detecting the relation between LDH level and the prognosis of COVID-19 patients, LDH level was taken as both classified variable and continuous variable in the regression analyses. Based on the result of regression analyses, Kaplan-Meier survival analyses were used to explore whether LDH levels were associated with prognosis. Curve fitting analysis was performed to assess the relation between CT performances and survival time. Two-sided *p*-values < 0.05 were regarded as statistically significant.

## Results

### Demographics, Clinical Information, and Treatment

The mean age of the patients in the elevated LDH group was 63.66 ± 14.49 years, which was higher than that in the normal/decreased LDH group (57.51 ± 14.36, *P* = 0.002; Table 1). Severe and critical cases account for a major part of patients with elevated LDH level no matter on admission or in the highest level of illness severity during hospitalization (both *P* < 0.001; Table 1). In addition, more patients in the elevated LDH group required critical airway management [tracheal intubation or extracorporeal membrane oxygenation (ECMO)] (*P* < 0.001; Table 1), and those in the elevated LDH group had significantly higher in-hospital mortality (12.7%) than those in the normal/decreased LDH group (0.50%, *P* < 0.001; Table 1). Patients in the elevated LDH group were also more likely to need anticoagulation treatment and corticosteroids (both *P* < 0.001; Table 1).

**Table 1:** Demographic and clinical information for COVID-19 patients in different LDH level.

**Covariate**	**LDH normal or decreased group** **(***n*** = 1,696)**		**LDH elevated group** **(***n*** = 55)**		***P*** **-value**
Age, year, mean ± SD	57.51 ± 14.36		63.66 ± 14.49		0.002
**Sex**					
Female	890	52.50%	26	47.30%	0.447
Male	806	47.50%	29	52.70%	
Comorbidity	487	60.30%	36	73.50%	0.066
Cardiovascular disease	331	41.00%	22	44.90%	0.587
Pulmonary disease	82	10.60%	5	13.90%	0.533
Nervous system disease	50	6.20%	4	8.20%	0.581
Endocrine disease	129	16.00%	6	12.20%	0.488
Malignancy	56	6.90%	3	6.10%	0.828
Digestive system disease	41	5.10%	4	8.20%	0.347
**Illness status of COVID-19 on admission**					
Mild	649	38.30%	14	25.50%	<0.001
Common	770	45.40%	14	25.50%	
Severe	260	15.30%	20	36.40%	
Critical	17	1.00%	7	12.70%	
**The highest level of illness status at hospitalization**					
Mild and common	903	53.40%	5	9.30%	<0.001
Severe	756	44.70%	36	66.70%	
Critical	33	2.00%	13	24.10%	
**The highest level of oxygen support**					
Low flow oxygen therapy	250	86.20%	6	33.30%	<0.001
High flow oxygen therapy	39	13.40%	7	38.90%	
Tracheal intubation	1	0.30%	4	22.20%	
ECMO	0	0.00%	1	5.60%	
**Symptoms when admitted to the hospital**					
Fever or myalgia	575	79.00%	35	79.50%	0.929
Respiratory system symptoms	588	80.80%	35	79.50%	0.842
Digestive system symptoms	74	10.20%	6	13.60%	0.444
Nervous system symptoms	24	3.30%	2	4.50%	0.655
CT score 1 in the first time	2.31 ± 0.71		2.58 ± 0.72		0.084
CT score 2 in the first time	2.30 ± 0.78		2.63 ± 0.65		0.053
CT total score in the first time	4.62 ± 1.28		5.21 ± 1.10		0.035
Antiviral therapy	811	99.10%	41	100.00%	0.552
Antibiotic therapy	484	99.40%	34	100.00%	0.646
Anticoagulation treatment	103	6.10%	21	38.20%	<0.001
Use of corticosteroid	90	5.30%	17	30.90%	<0.001
Death	8	0.50%	7	12.70%	<0.001
Follow-up days, mean ± SD	19.26 ± 8.893		22.96 ± 10.38		0.004

### Laboratory Findings

As shown in Table 2, there were significant differences in most laboratory indexes according to the LDH level. The median interleukin-6 in the elevated LDH group was above the normal range and was significantly higher than that in the normal/decreased LDH group, as was D-dimer, indicating that patients with elevated LDH had a more intense inflammatory responses and more of these patients were in a hypercoagulable state. In addition, patients with elevated LDH were more likely to have lymphopenia (*P* < 0.001). However, the prevalence of SARS-CoV-2 immunoglobulin M and immunoglobulin G did not differ significantly according to the LDH level.

**Table 2:** Outcomes of laboratory tests for COVID-19 patients in different LDH level.

**Covariate**	**LDH normal or decreased group** **(***n*** = 1,696)**	**LDH evaluated group** **(***n*** = 55)**	***P*** **-value**	**Reference**
Interleukin-6, pg/mL	1.50 (1.50–3.74)	17.77 (4.17–49.24)	<0.001	0.00–7.00
Procalcitonin, ng/mL	0.04 (0.03–0.05)	0.11 (0.06–0.21)	<0.001	<0.05
Alanine aminotransferase, U/L	22.00 (15.00–36.00)	44.00 (29.00–80.00)	<0.001	9.00–50.00
Aspartate aminotransferase, U/L	19.00 (16.00–26.00)	42.60 (31.00–86.00)	<0.001	15.00–40.00
Albumin, g/L	37.80 (35.10–40.10)	32.80 (30.30–36.20)	<0.001	40.00–55.00
Creatine kinase, ng/mL	51.00 (36.00–74.00)	91.00 (50.00–172.00)	<0.001	18.00–198.00
Total bilirubin, μmol/L	9.10 (7.00–11.90)	9.50 (6.00–18.00)	0.344	5.00–21.00
Direct bilirubin, μmol/L	3.10 (2.40–4.20)	4.60 (2.60–8.00)	<0.001	0.00–7.00
Indirect bilirubin, μmol/L	5.70 (4.30–7.80)	4.80 (3.40–7.60)	0.085	1.50–1.80
Creatinine, μmol/L	64.10 (54.10–76.00)	67.10 (57.40–90.00)	0.730	64.00–104.00
Ureanitrogen, mmol/L	4.80 (3.90–5.80)	5.90 (4.10–8.19)	0.001	2.80–7.60
INR	0.97 (0.93–1.01)	1.01 (0.97–1.10)	<0.001	0.85–1.15
Prothrombin time, s	11.30 (10.90–11.70)	11.7 (11.3–12.73)	<0.001	9.40–12.50
Thrombin time, s	17.60 (17.00–18.30)	16.95 (16.23–18.00)	0.001	10.30–16.60
Activated partial thromboplastin time, s	27.20 (24.55–30.40)	28.20 (24.98–33.13)	0.095	25.10–36.50
Fibrinogen, g/L	2.92 (2.51–3.67)	4.08 (3.09–4.75)	<0.001	2.38–4.98
D-dimer, mg/L	0.37 (0.21–0.87)	1.37 (0.61–4.07)	<0.001	<0.50
White blood cell count, × 10^9^/L	5.68 (4.70–6.8)	6.92 (5.27–9.40)	<0.001	3.5–9.5
Neutrophil count, × 10^9^/L	3.25 (2.53–4.23)	4.53 (3.35–7.89)	<0.001	1.8–6.3
Lymphocyte count, × 10^9^/L	1.62 (1.27–1.99)	0.96 (0.59–1.35)	<0.001	1.1–3.2
Monocyte count, × 10^9^/L	0.50 (0.40–0.63)	0.57 (0.41–0.74)	0.108	0.1–0.6
Red blood cell count, × 10^9^/L	4.12 (3.77–4.49)	3.90 (3.52–4.40)	0.035	4.3–5.8
Hemoglobin, g/L	126.00 (115.00–137.00)	122.00 (107.00–134.00)	0.125	130–175
Hematocrit, %	38.00 (34.90–40.90)	36.90 (32.20–40.20)	0.041	40.00–50.00
Platelet count, × 10^9^/L	229.00 (188.00–277.00)	203.00 (141.00–280.00)	0.020	125.00–350.00
IgM (+) of SARS-CoV-2	199 (35.20%)	9 (40.90%)	0.584	(–)
IgG (+) of SARS-CoV-2	504 (29.70%)	19 (34.50%)	0.316	(–)

### Analysis for the Relationship Between Prognosis and LDH Level

The univariate Cox regression analysis showed that patients in the elevated LDH group had a higher risk of in-hospital death than those in the normal or decreased LDH group [hazard ratio (HR): 26.626, 95% confidence interval (CI): 9.624–73.661, *P* < 0.001; Table 3]. The result of univariate logistics regression analysis presented the same tendency that elevated LDH group suffered higher risk of developing into sever or critical illness status than those in the normal or decreased LDH group [odds ratio (OR): 11.216, 95% CI: 4.447–28.288, *P* < 0.001; Supplementary Table 1]. The adjustment factors included in the multivariate Cox regression model were age, history of cardiovascular disease, white blood cell count, platelet count, lymphocyte count, and D-dimer. The results of the multivariate analysis showed that an elevated LDH level was an independent risk factor for in-hospital death (*P* = 0.024; Table 3). Elevated LDH level was not related to sever or critical illness status after adjustment (*P* = 0.997; Supplementary Table 1).

**Table 3:** Univariate and multivariate Cox regression analysis for the survival of patients in different LDH level.

**Group**		**Cox regression analysis**			
		**HR**	**95% CI**		***P*** **-value**
Univariate analysis	LDH normal or decrease group	ref			
	LDH evaluated group	26.626	9.624	73.661	<0.001
Multivariate analysis^*^	LDH normal or decrease group	ref			
	LDH evaluated group	4.491	1.218	16.560	0.024

* *Adjust for age, the history of cardiovascular disease, WBC, PLT, lymphocyte count, D-Dimer*.

LDH was taken as a continuous variable in further analysis. The result was similar to the previous analysis which LDH level was divided into groups. LDH was an independent risk factor for in-hospital death (univariate analysis *P* < 0.001, multivariate analysis *P* = 0.001; Table 4) but not for severe or critical illness status (univariate analysis P < 0.001, multivariate analysis *P* = 0.557; Table 4). Each unit increase in LDH level was associated with higher risk of death (univariate analysis: HR = 1.002, 95 CI%: 1.001–1.002; multivariate analysis: HR = 1.006, 95 CI%: 1.002–1.009).

**Table 4:** Univariate and multivariate cox regression analysis for the survival of patients and logistics regression analysis for the severity of patients when taking LDH as a continuous variable.

**Group**		**Cox regression analysis**				**Logistics regression analysis**			
		**HR**	**95% CI**		***P*** **-value**	**OR**	**95% CI**		***P*** **-value**
Univariate analysis	LDH level	1.002	1.001	1.002	<0.001	1.012	1.010	1.014	<0.001
Multivariate analysis^*^	LDH level	1.006	1.002	1.009	0.001	1.003	0.992	1.014	0.577

* *Adjust for age, the history of cardiovascular disease, WBC, PLT, lymphocyte count, D-Dimer*.

**Figure 1:**
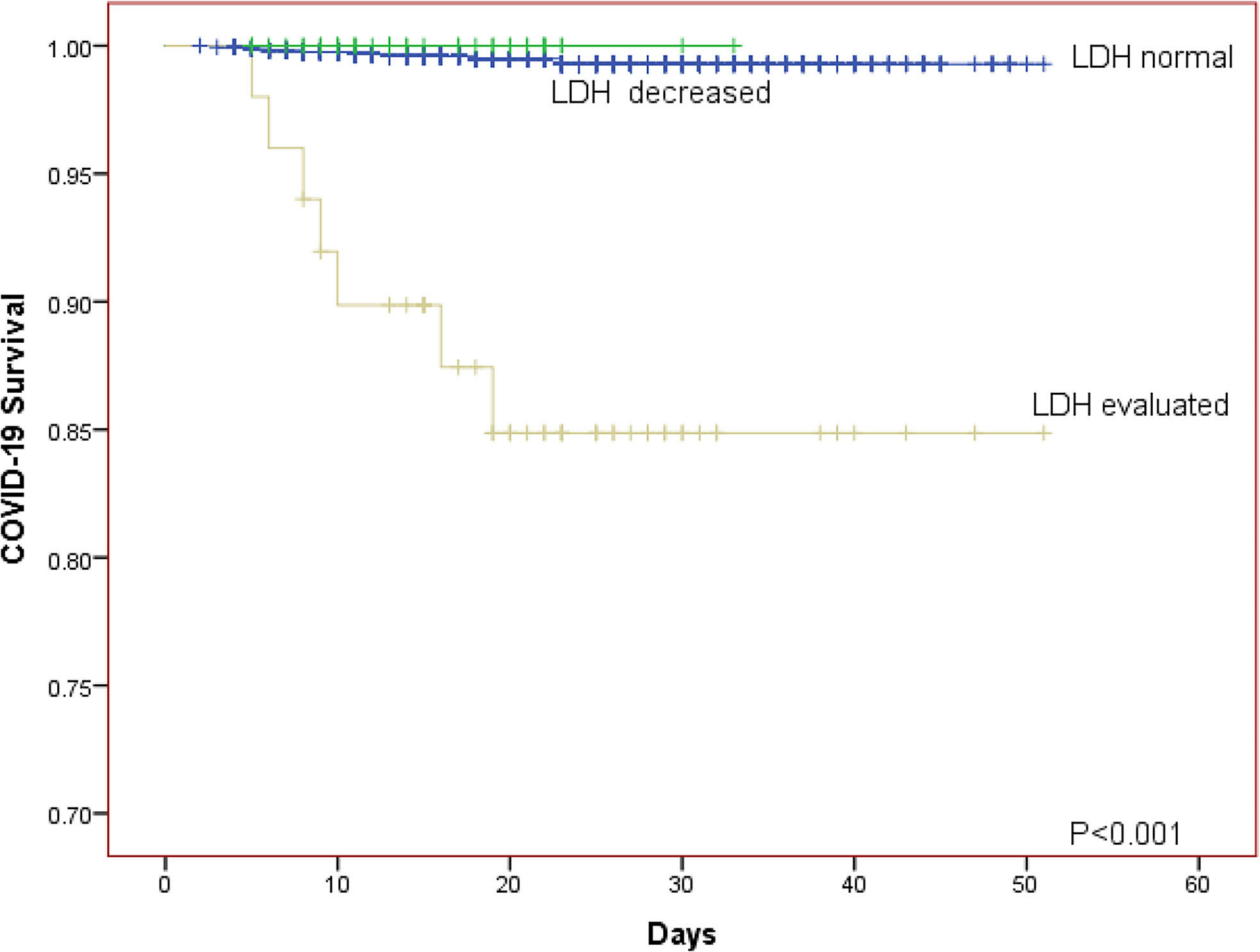
The Kaplan-Meier curves for the survival of COVID-19 patients in different LDH level (normal group, decreased group, evaluated group).

**Figure 2:**
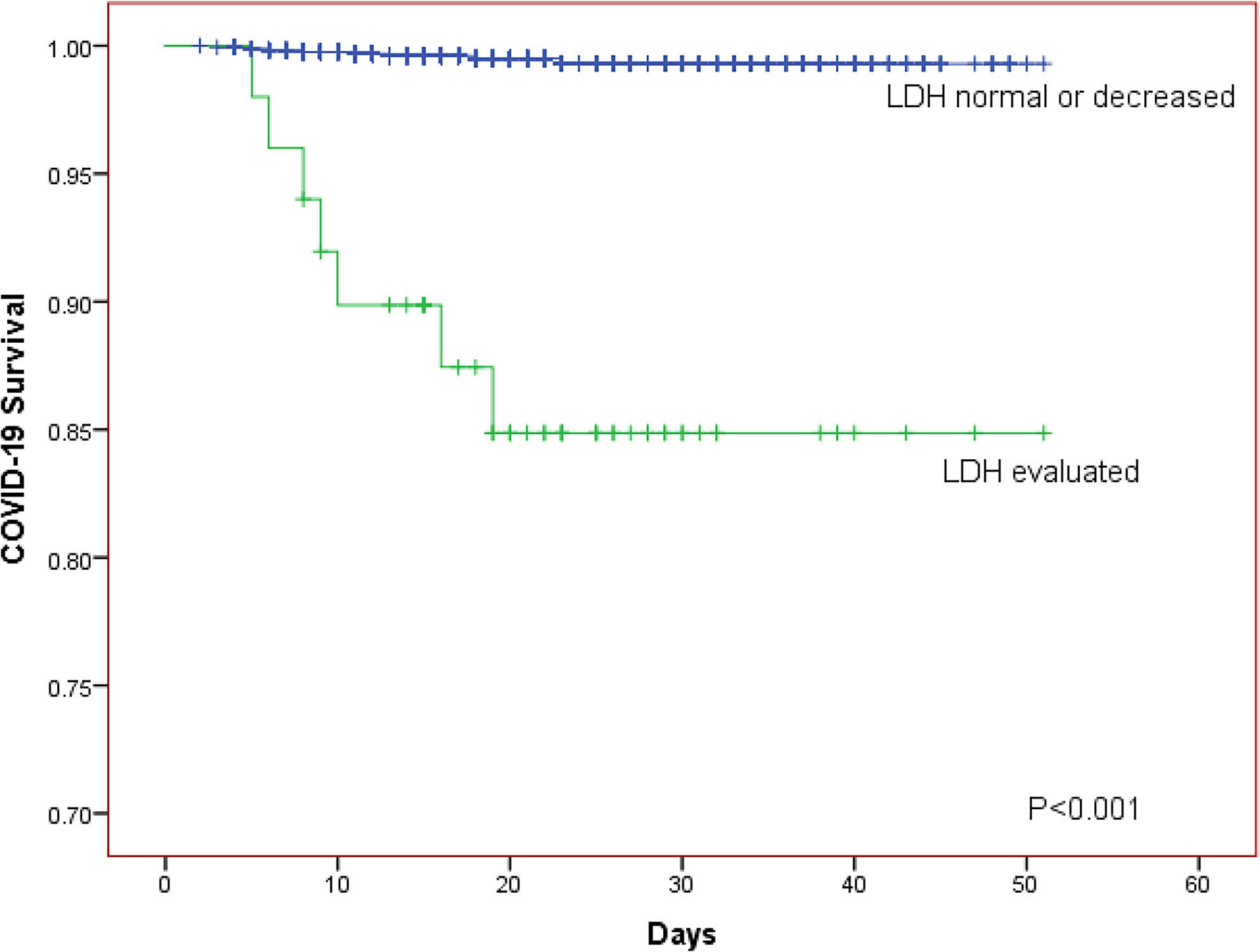
The Kaplan-Meier curves for the survival of COVID-19 patients in different LDH level (normal or decreased group, evaluated group).

The Kaplan-Meier curves was further generated for descripting the relationship between survival of patients and LDH level. The Kaplan-Meier curves for LDH level showed that patients in the elevated LDH group had worse prognosis than those with normal/decreased LDH regardless of whether the patients were divided into two or three LDH groups (both *P* < 0.001; Figures 1, 2).

### Curve Fitting Analysis for the Evaluation of CT Images

**Figure 3:**
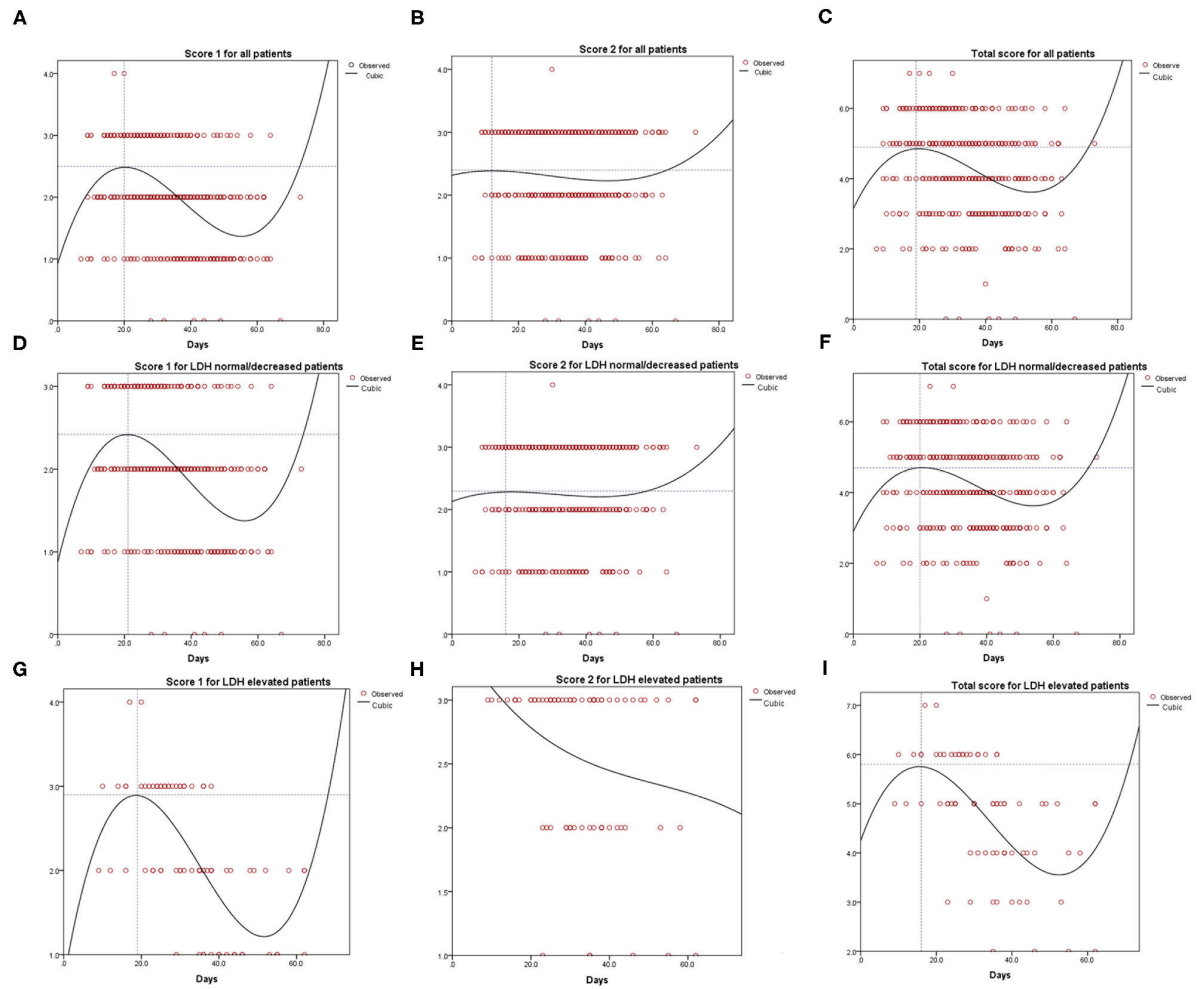
Curve fitting analysis for all the COVID-19 patients **(A–C)**, LDH normal or decreased group **(D–F)** and LDH evaluated group **(G–I)**.

Figure 3 shows the result of the curve fitting analysis for CT images and days that the CT scan was done. Score 1 for all patients reached the peak at 20 days (2.5 points, Figure 3A), while score 2 for all patients reached the lowest point at 12 days (2.40 points, Figure 3B). The total score reached the peak at 19 days (4.90 points, Figure 3C). Similarly, score 1 for patients with normal/decreased LDH reached the peak of 2.42 at 21 days (Figure 3D), score 2 reached the peak of 2.30 at 16 days (Figure 3E) and total score reached the peak of 4.70 at 20 days (Figure 3F). For patients with evaluated level of LDH, score 1, and total score reached the peak of 2.90, 5.80 on 19, 16 days, respectively (Figures 3G,I). However, the tendency of score 2 for patients with elevated LDH level tended to be descending (Figure 3H).

## Discussion

We investigated the effect of LDH on the clinical course and survival of patients with laboratory-confirmed COVID-19 based on a large sample with 1,880 patients and found that patients with elevated LDH were associated with higher mortality on univariate or multivariate Cox regression analysis no matter LDH was taken as classified variable or continuous variable. The Kaplan-Meier curves for COVID-19 progress also showed the same tendency.

In addition to the reticulocyte count, indirect bilirubin levels, serum haptoglobin, and LDH levels have been used as markers of hemolysis (5). In another study, Tasaka et al. (12) found that measuring LDH levels could help improve the diagnosis of pneumocystis pneumonia. Furthermore, they found that the HIV-positive patients had higher LDH levels than HIV-negative patients. These studies reveal that LDH plays an important role in differentiating disease, including that of the immune system. In our study, although we did not compare the LDH levels in patients with COVID-19 with the LDH levels in patents with other types of pneumonia or the normal population, an elevated LDH level was predictive of higher mortality in patients with COVID-19. Therefore, LDH was shown to be associated with disease diagnosis and prognosis.

COVID-19 patients with higher LDH levels tended to be older, and were more likely to require respiratory support. On the other hand, the patients in the elevated LDH group had similar comorbidities to the other patients. In patients with pneumonia, the presence of comorbidities may adversely affect the clinical course and the outcome (13). In our cohort, the prevalence of pulmonary disease did not differ according to the LDH level; therefore, the comorbidities did not act as confounders of the association between LDH levels and survival in patients with COVID-19. Previous study found that the levels of LDH in severe cases of COVID-19 were significantly higher than both non-severe cases of COVID-19 and healthy control group, while the LDH level of non-severe cases were also higher than healthy group (14). In this study, LDH level was associated with severe or critical illness status in univariate logistics regression analysis, which was in accordance with the result of previous study. However, significant differences were not found in multivariate regression analysis which contained adjustment of confounding factors including age, the history of cardiovascular disease, WBC, PLT, lymphocyte count, D-Dimer. We hypothesized that elder patients and patients with the history of cardiovascular disease essentially burden higher risk of cardiac muscle or lung interstitial damage.

In the early phase of COVID-19, CT images reveal multifocal peripheral and basal ground-glass opacities, crazy paving patterns, traction bronchiectasis, and air bronchogram signs (15, 16). However, depending on various factors, such as the evolution of the disease course or severity, comorbidity and therapy, CT presentations are dynamic and manifestation patterns often overlap (13, 17–19). With the progression of the clinical course, the CT manifestations include pleural effusion, irregular interlobular, and septal thickening (16). In this study, the CT manifestations were evaluated and presented as score 1 (imaging feature type), score 2 (lesion distribution), and the total score (score 1 plus score 2) by two independent radiologists to record the dynamic changes (20). Fitting curve for imaging manifestations types of lung inflammation and lesion distribution in normal or lower LDH group showed a trend of first rise then descend, however, higher LDH group patients showed a trend of rapidly rising and then rapidly falling (Figures 3G,I) or presented as a trend of declining all along (Figure 3H). This may be because patients with elevated LDH tended to have severe clinical symptoms of pneumonia and were then transported to the hospital for unified and timely medical treatment.

In addition, the use of antiviral therapy and antibiotic therapy did not differ according to the LDH level among the patients in our study. However, a higher proportion of patients with elevated LDH received anticoagulation treatment and corticosteroid. Drug treatment, especially the use of corticosteroids, may slow virus clearance due to its immunosuppressive effect (21, 22). This may affect the disease course and biochemical indicators, including LDH; therefore, further research is needed to determine the effects of corticosteroids and anticoagulants on LDH in patients with COVID-19.

Other studies have found that an elevated LDH level is a sensitive biomarker for lymphoproliferative disorders (23, 24). Ghobrial et al. (25) and Boothpur et al. (26) identified that serum LDH was one of the negative prognostic factors for overall survival and recurrence. Many studies have found a significant drop in T lymphocyte subsets and an increase in inflammatory cytokines in patients with COVID-19 (8, 27). Nguyen et al. (24) demonstrated that the SARS-CoV-2 virus could enable cross-protective T-cell based immunity in a comprehensive *in silico* analysis. In our study, patients in the elevated LDH group had a higher white blood cell count but lower lymphocyte count than patients with normal/decreased LDH. Overwhelming inflammation and cytokine-associated lung injury could be important factors in initiating severe events in patients with COVID-19 (28), Therefore, LDH may affect the clinical course of COVID-19 by causing inflammation and lung injury, and influencing T-cell based immunity.

Jiang et al. found that patients with COVID-19 had IgG and IgM antibodies which specifically combine with SARS-CoV-2 proteins, particularly the N protein and S1 protein (29). They also found that S1 specific IgG signal positively correlates with the level of LDH (29). However, whether serum lactate dehydrogenase have any similar physiological function or pathological pathway to affect the clearance of SARS-CoV-2 remains unclear and warrants further study.

This study has some limitations. Heterogeneity is an unavoidable limitation of retrospective studies Data of patients were collected retrospectively, which inevitably led to biases in our study. Another limitation is a lack of research on the mechanism of serum lactate dehydrogenase levels as a common risk factor for COVID-19 progress and prognosis. In addition, the role of drug interference such as glucocorticoids, antiviral and antibacterial treatment cannot be excluded. Further multi-center prospective studies with a larger sample size are needed to verify the findings and to determine the pathogenic mechanism by which LDH exerts an effect on patients with COVID-19.

## Conclusion

Our study revealed that LDH level is an independent risk factor for the survival of patients with COVID-19 and a high LDH level is a predictor of mortality in patients with COVID-19. However, LDH level seems not to be associated with severe or critical illness status. This study will provide a valuable reference for clinicians and researchers to understand, diagnose, and treat patients with COVID-19, although prospective studies with a larger sample size are needed to verify the findings and to determine the pathogenic mechanism by which LDH exerts an effect on patients with COVID-19.

## Data Availability Statement

The original contributions presented in the study are included in the article/Supplementary Material, further inquiries can be directed to the corresponding author/s.

## Ethics Statement

This study obtained the approval of the Research Ethics Commission of the Zhongnan Hospital of Wuhan University (approval number: 2020074). The requirement for informed consent was waived because the study was retrospective.

## Author Contributions

YH, ZLiu, and JL: conception and design. LG, KLu, and XW: administrative support. KLi and ZX: provision of study materials or patients. QW and JC: collection and assembly of data. YH, CZ, and ZLi: data analysis and interpretation. YH and ZLiu: manuscript writing. MW, WY, and XW: final approval of manuscript. All authors contributed to the article and approved the submitted version.

## Conflict of Interest

The authors declare that the research was conducted in the absence of any commercial or financial relationships that could be construed as a potential conflict of interest.

## Publisher's Note

All claims expressed in this article are solely those of the authors and do not necessarily represent those of their affiliated organizations, or those of the publisher, the editors and the reviewers. Any product that may be evaluated in this article, or claim that may be made by its manufacturer, is not guaranteed or endorsed by the publisher.
