# Supplementary material for: Serum Lactate Dehydrogenase Level as a Prognostic Factor for COVID-19: A Retrospective Study Based on a Large Sample Size
Source: Front Med (Lausanne). 2022 Jan 4;8:671667. doi: 10.3389/fmed.2021.671667 (PMC8763698; doi:10.3389/fmed.2021.671667)
Supplement: Supplementary file 1 [file Table_1.DOCX]

| Supplemental Table 1: Univariate and multivariate Logistics regression analysis for the severity of patients in different LDH level. | | | | | | | | |
| --- | --- | --- | --- | --- | --- | --- | --- | --- |
| **Group** | | **Logistics Regression Analysis** | | | |  |  |  |
|  |  | **OR** | **95 % CI** | | **P value** |  |  |  |
| Univariate Analysis | LDH normal or decrease group | ref |  |  |  |  |  |  |
|  | LDH evaluated group | 11.216 | 4.447 | 28.288 | <0.001 |  |  |  |
| Multivariate Analysis* | LDH normal or decrease group | ref |  |  |  |  |  |  |
|  | LDH evaluated group | 1.03E+07 | - | - | 0.997 |  |  |  |
| *Adjust for age, the history of cardiovascular disease, WBC, PLT, lymphocyte count, D-Dimer | | | | | | | | |

Curve fitting equations for Figure 3：

y=A+z(1)*x+z(2)*x^2^+z(3)*x^3^ , x= time in hospitalization

A, z(1), z(2), z(3) for each curve in Figure 3 are presented in the table below.

|  |  |  |  |  |
| --- | --- | --- | --- | --- |
| Figure 3 | A | z(1) | z(2) | z(3) |
| a | 0.92 | 0.18 | -0.01 | 5.23E-05 |
| b | 2.31 | 0.01 | 0.00 | 7.70E-06 |
| c | 3.15 | 0.20 | -0.01 | 6.20E-05 |
| d | 0.87 | 0.17 | -0.01 | 4.81E-05 |
| e | 2.13 | 0.02 | 0.00 | 8.54E-06 |
| f | 2.90 | 0.20 | -0.01 | 5.90E-05 |
| g | 0.69 | 0.27 | -0.01 | 9.34E-05 |
| h | 3.56 | -0.05 | 0.00 | -5.71E-06 |
| i | 4.25 | 0.22 | -0.01 | 8.77E-05 |
|  |  |  |  |  |
